# The pectoralis minor length test: a study of the intra-rater reliability and diagnostic accuracy in subjects with and without shoulder symptoms

**DOI:** 10.1186/1471-2474-8-64

**Published:** 2007-07-09

**Authors:** Jeremy S Lewis, Rachel E Valentine

**Affiliations:** 1 Therapy Department, Chelsea and Westminster Hospital NHS Foundation Trust, 369 Fulham Road, London, SW10 9NH, UK; 2 Therapy Department, St George's Hospital, London, UK

## Abstract

**Background:**

Postural abnormality and muscle imbalance are thought to contribute to pain and a loss of normal function in the upper body. A shortened pectoralis minor muscle is commonly identified as part of this imbalance. Clinical tests have been recommended to test for shortening of this muscle. The aim of this study was to evaluate the intra-rater reliability and diagnostic accuracy of the pectoralis minor length test.

**Methods:**

Measurements were made in 45 subjects with and 45 subjects without shoulder symptoms. Measurements were made with the subjects lying in supine. In this position the linear distance from the treatment table to the posterior aspect of the acromion was measured on two occasions (separated by a minimum of 30 minutes and additional data collection on other subjects to reduce bias) by one rater. The reliability of the measurements was analyzed using intraclass correlation coefficients (ICC), 95% confidence intervals (CI) and standard error of measurement (SEM). The diagnostic accuracy of the test was investigated by determining the sensitivity, specificity, positive and negative likelihood ratios of the test against a 'gold standard' reference. The assessor remained 'blinded' to data input and the measurements were staggered to reduce examiner bias.

**Results:**

The pectoralis minor length test was found to have excellent intra-rater reliability for dominant and non-dominant side of the subjects without symptoms, and for the painfree and painful side of the subjects with symptoms. The values calculated for the sensitivity, specificity, positive and negative likelihood ratios suggest this test performed in the manner investigated in this study and recommended in the literature, lacks diagnostic accuracy.

**Conclusion:**

The findings of this study suggest that although the pectoralis minor length test demonstrates acceptable clinical reliability, its lack of specificity suggests that clinicians using this test to inform the clinical reasoning process with regard treatment planning must do so with caution.

**Trial registration:**

National Research Register: N0060148286

## Background

Musculoskeletal disorders of the shoulder are extremely common, with 1 in 3 people experiencing shoulder pain at some stage of their lives [1]. Shoulder pathology is the third most common musculoskeletal condition treated in primary care and up to 2% of the population consult with their General Practitioner annually because of pain and dysfunction in this region [2-5]. Of concern, shoulder pathology is associated with substantial dysfunction and morbidity [6], with 54% of sufferers reporting on-going symptoms after 3 years [7].

In the absence of a specific or identifiable cause of symptoms, poor upper body posture, colloquially referred to as a 'forward head posture', 'slouched posture', 'poking chin posture', or 'rounded shoulder posture' has been cited as a potential etiological factor in the pathogenesis and perpetuation of many clinical syndromes involving the shoulder. Beliefs relating to posture have permeated into clinical practice and are frequently used to explain to patients the basis for pathology and the rational for rehabilitation. An example of this is provided by Gray and Grimsby [8] (p138):

"In a person with good postural alignment, elevation of the arm is free to proceed through a full 160° to 180° of motion without impingement of soft tissues in the subacromial space. In the patient with the classic forward head, rounded shoulders, and increased thoracic kyphosis, the scapula rotates forward and downward, depressing the acromial process and changing the direction of the glenoid fossa. Now as the patient attempts to elevate the arm, the supraspinatus tendon and/or the subdeltoid bursa may become impinged against the anterior portion of the acromion process. Repeat motions of this nature may accelerate overuse injuries or cumulative trauma disorders and lead to early changes consistent with tendinitis and/or bursitis".

These theories are appealing and the suggestion that once a postural abnormality is identified, restoration to an ideal 'normal' posture will lead to a reduction in symptoms and improvement in function. In the specific example given above [8] the rationale is that an improvement in shoulder posture will lead to a reduction of the impingement process, reduced irritation on the rotator cuff tendons and a lessening in symptoms. Within the physical therapy, osteopathic and medical literature there is considerable reference to faulty posture and muscle imbalance and its relationship with shoulder pathology and symptoms. In addition to this, clinical assessment and rehabilitation procedures have been proposed to identify and treat postural abnormalities [9-17].

One muscle that is frequently implicated in shoulder and upper quadrant pathology is the pectoralis minor and a shortening of this muscle has been associated with a forward head posture [9-11,16,18,19].

It has been argued that due to its attachment on the coracoid process a shortening of pectoralis minor will lead to the anterior tilting of the scapula [11,16,20]. Sahrmann [16] has described a number of clinical syndromes that are associated with a shortening of pectoralis minor. These include; thoracic outlet syndrome, scapular winging and tilting syndrome, scapular abduction syndrome, scapular depression syndrome and scapular downward rotation syndrome. To identify a postural shortening of pectoralis minor in association with these and other upper quadrant syndromes a test of the muscles length has been proposed. Sahrmann [16] (p211 [Figure 5–30] and p338–340) has described that when the pectoralis minor muscle is of normal length the distance between the treatment table and posterior aspect of the acromion (patient supine, arms by side, elbows flexed) should not exceed 2.54 cm (1 inch). A distance greater than this would suggest a muscle imbalance had occurred and the muscle had shortened. Identifying a muscle imbalance involving a short pectoralis minor is then used within the context of the clinical reasoning process to inform and direct the clinician as regards appropriate therapeutic intervention.

Although posture and muscle imbalance is commonly implicated as part of the pathological process the evidence available to support these theories is limited, with research studies reporting equivocal findings [21-27]. In addition to this, the concept of correcting posture and its associated muscle imbalance through stretching and strengthening programs [16,28] has been widely accepted. However, there is no definitive evidence that an ideal posture exists [29], or that deviations from an ideal norm are associated with compromised function and disability [12,22,23,30]. As clinical practice is frequently based on the assessment of posture and muscle imbalance it is important that fundamental questions concerning these issues are addressed.

To be of value, clinical tests must be reliable and have acceptable diagnostic accuracy. In part, the findings of clinical tests and measurements are used by clinicians to inform the clinical reasoning process. To have meaning these tests must be reliable. Sim and Wright [31] have defined reliability in three categories; equivalence, stability and internal consistency. Internal consistency relates to the homogeneity of a multi-item instrument. Equivalence relates to the consistency of measurements, for a given entity, when used by two or more investigators. Stability relates to the consistency of an instrument when used to measure the same entity on repeated occasions. Equivalence and stability are sometimes respectively referred to as reproducibility and repeatability [31,32]. Stability (repeatability) is usually determined for a single investigator and is generally referred to as intra-rater reliability and is sometimes referred to as test-retest reliability [31]. Most clinical tests have two possible outcomes. A positive test implies the condition is present and a negative result implies the condition is not present. This may be expressed by the sensitivity and specificity values for a test. An additional method of describing the diagnostic value of a test includes the positive and negative likelihood ratios. Likelihood ratios provide numerical information concerning the likelihood that a test result or finding would be present in a patient with the disorder or condition in comparison to the likelihood that the same finding or result would be expected in a patient without the condition or disorder [33]. In addition, likelihood ratios provide a robust determination of the usefulness of a clinical test as they incorporate both the sensitivity and specificity together in one analysis and do not treat them as separate entities.

To determine the diagnostic accuracy of a test the clinical measurements are compared to a 'gold standard' reference test. At present there is no gold standard reference test for the measurement of pectoralis minor length. Sahrmann [16] (p211) has stated that the shoulders tilt anteriorly because of a shortness of pectoralis minor and that the lateral border of the spine (posterior aspect of the acromion) should be no more than 2.54 (2.6) cm from the treatment table when the subject is in supine. For the purposes of this investigation and to attempt to establish a relationship between pectoralis minor length and symptoms a negative pectoralis minor length test was defined as a table to posterior acromion measurement of less than or equal to 2.6 cm, and a positive test as being a measurement greater then 2.6 cm.

A review of the medical data bases (MEDLINE, CINAHL, AMED, PEDro, EMBASE) and a manual literature search; using the search terms; pectoralis minor, length, length test, posture, forward head posture, scapular position, scapula, shoulder, diagnosis, and reliability failed to identify any English language publication that has investigated the reliability of the pectoralis minor length test in subjects with and without shoulder symptoms and the diagnostic accuracy of the test in subjects with shoulder symptoms.

Therefore the aims of this investigation were to determine the;

(i) intra-rater reliability of the pectoralis minor length test in subjects without symptoms

(ii) intra-rater reliability of the pectoralis minor length test in subjects with symptoms, and

(iii) diagnostic accuracy of this clinical test of pectoralis minor length against the 'gold standard' recommendation of normal range being no greater than 2.6 cm above the table.

## Methods

### Subjects

Subjects with symptoms were recruited through the orthopedic and physical therapy out-patient department in the teaching hospital where the study was conducted. Subjects without symptoms were recruited through personal and public advertisements. Permission to conduct this study was granted by the local research ethics committee. All subjects signed witnessed informed consent documents and were aware of all their rights including the right to withdraw from the study at any stage of the investigation.

### Inclusion/exclusion criteria

Inclusion criteria for the subjects with symptoms were; unilateral pain and/or restriction of movement arising from the area of the shoulder (C4/C5 dermatome). Inclusion criteria for the subjects without symptoms were; no lumbar, thoracic, cervical or shoulder or upper limb symptoms. Exclusion criteria for both groups were; an inability to fully communicate in English, subjects younger than 18 years of age, cardiac, respiratory, kidney, circulatory problems, systemic disease, diabetes, pregnancy, and, for female subjects pregnancy or suspicion of pregnancy. For subjects without symptoms additional exclusion criteria were; a history of fractures, treatment or surgery to the lumbar, thoracic, cervical spine and upper limbs.

### Procedure

**Figure 1:**
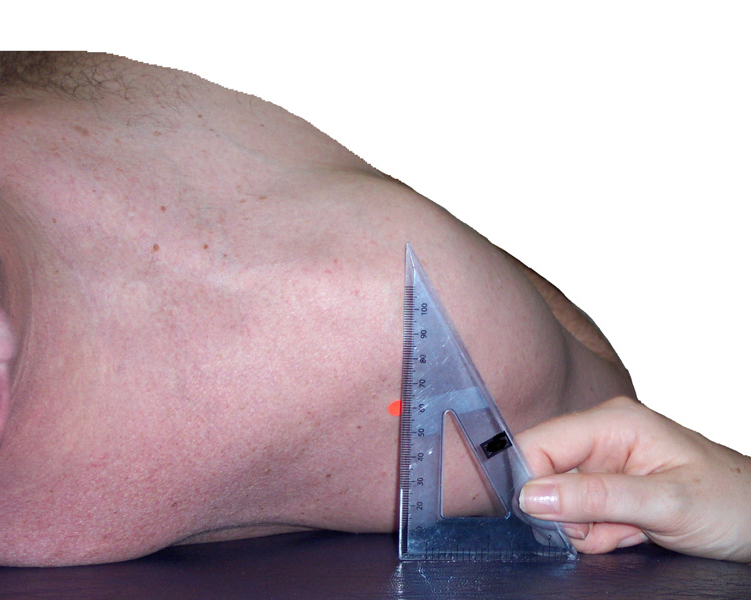
The pectoralis minor length test measurement.

This investigation involved measuring the linear distance from the treatment table to the posterior aspect of the acromion in subjects with and without symptoms. Subjects were requested to lie supine on a standard treatment table and adopt their natural relaxed posture. As described by Sahrmann [16] the subjects placed their arms by their sides and the elbows were flexed and rested against the lateral wall of the abdomen. The subject's hands rested gently on the abdomen which would have placed the glenohumeral joint in slight internal rotation. Random number tables were used to randomly allocate subjects to the side to be tested first. The investigator measured the linear distance in millimeters using a rigid standard plastic transparent right angle (WH Smith PLC, 180 Wardour Street, London W1F 8FY, UK) with a height of 12 cm and a base of 8 cm. Without exerting any downward pressure into the table, the base of the protractor was placed on the treatment table and the vertical side was placed adjacent to the lateral aspect of the acromion. It was observed by the researchers that the distance (as measured with the plastic right angle) from the treatment table to the posterior acromion did not change if the subjects arms were by the side, actively held by the side with the fingers pointing to the ceiling or resting as described above gently on the abdomen. In addition to this attention to ensure that the plastic right angle did not bend during the investigation was carefully adhered to. The measuring technique is detailed in Figure 1.

Each measurement for each side was measured 3 times in succession, and on each occasion the right angle was replaced as previously described. The investigator verbally relayed the measurements to an assistant who recorded each set of measurements on an individualized data entry sheet. At all stages the investigator was blinded to data entry. An interval of approximately 30 minutes separated the two sets of measurements made on each subject. This interval served to reduce examiner bias to ensure the investigator was unable to recall any earlier measurements. In addition to this, and to further reduce the investigators ability to recall any measurements, the data collection protocol was staggered in the following way; subject 1 (first set of measurements), subject 2 (first set of measurements), subject 1 (second set of measurements), subject 2 (second set of measurements), etc.

### Power analysis

This study formed part of a series of studies aiming to investigate measurement reliability as well as relationships of posture for the shoulder and upper body. Walter et al [34] have provided estimates for sample size requirements for reliability studies using intraclass correlation coefficients (ICC). For a true *p*0 of .7 against an alternative *p*1 of .9, based on a 5% significance level and a power of 80% (β = .20) for two raters, or two time points, 19 subjects are required [31,34]. Forty five subjects were recruited into each group (90 in total). This number of subjects was considered adequate to determine the intra-rater reliability for measuring the linear measurement of interest in this study. Forty-six subjects are the required number for a true *p*0 of .8 against an alternative *p*1 of .9 [34].

### Statistical analysis

The reliability of the measurements was analyzed using intraclass correlation coefficients (ICC), 95% confidence intervals (CI) and standard error of measurement (SEM). The descriptive statistics, ICC (Model 2), 95% CI and the SEM statistics were analyzed using SPSS version 14 software (SPSS-UK Ltd, St. Andrews House, West Street, Woking, Surrey, GU21 6EB, United Kingdom). The analysis of reliability involved determining the reliability of (i) the first measurement and for (ii) the mean of the 3 measurements. Portney and Watkins [35] have described 6 different equations for calculating ICC, and has argued that Model 2 should be used when wishing to confidently generalize the findings of a reliability trial of a particular method of measurement to equally trained clinicians, and Model 3 should be selected when an investigator is interested in establishing the reliability of a measurement procedure for one specific data collection experience without the intention to generalize the findings to equally trained clinicians (Portney and Watkins [35] page 562). ICC (Model 2) was used in the current analysis. Using SPSS version 14 software, ICC Model (2,1) was analyzed by selecting the options; two-way random, single measure, absolute agreement and Model (2,3) was analyzed by selecting; two-way random, average measure, absolute agreement.

An ideal clinical test will both a have a sensitivity of 1 or 100% and specificity of 1 or 100%. An additional method of describing the diagnostic value of a test includes the positive and negative likelihood ratios. A positive likelihood ratio, or the ratio of the true positive rate to the false positive rate, provides an indication of how much more likely a positive test finding is in people with the condition than those without, and a negative likelihood ratio, or the ratio of the false positive rate to the true negative rate, provides an indication of how much more likely a negative test finding is for patients who have the condition, than those that don't. A positive likelihood ratio of greater than 10 suggests the test is very useful and a negative value of less than 0.1 is also very useful [33].

To determine the relationship between the pectoralis minor length test and symptoms a 2 × 2 table was constructed [33]. The upper left cell (a) contained the number of subjects with symptoms who had a pectoralis minor length test distance of greater than 2.6 cm (true positives). The upper right cell (b) contained the number of subjects without symptoms who had a pectoralis minor length test distance of greater than 2.6 cm (false positives). The bottom left cell (c) contained the number of subjects with symptoms with a pectoralis minor length test distance of equal to or less than 2.6 cm (false negatives) and the bottom right cells (d) contained the number of asymptomatic subjects with a pectoralis minor length test distance of equal to or less than 2.6 cm (true negatives).

## Results

Ninety subjects were recruited for this investigation. Of the 45 recruited in the group of subjects with symptoms 23 (51.1%) females and 22 male subjects (48.9%) and in the group without symptoms (n = 45) there were 24 (53.3%) female subjects and 21 male subjects (46.7%). Patients were referred from orthopaedic surgeons and general practitioners and the diagnoses written on the referral were recorded. The most common diagnoses for the symptomatic subjects were; non-specific shoulder pain (n = 21), and rotator cuff tendinopathy (n = 12). Other diagnoses included; frozen shoulder (n = 2), acromioclavicular joint pain (n = 2), glenohumeral instability (n = 2), stable humeral fractures (n = 1) and stable scapular fractures (n = 1). Further information is detailed in the following table (Table 1).

**Table 1:** Descriptive information for subjects with and without shoulder symptoms

	Subjects without symptoms			Subjects with symptoms		
	Age (years)	Height (m)	Weight (kg)	Age (years)	Height (m)	Weight (kg)
	
Mean (SD)	32.1 (7.3)	1.7 (0.1)	70.4 (14.2)	42.8 (16.6)	1.7 (0.1)	71.4 (11.8)
Range	23.0–56.0	1.58–1.91	50.0–111.0	19.0–84.0	1.49–1.90	49.0–90.0

Abbreviations: SD (standard deviation)

The mean table to posterior acromion distance for the (i) left and right shoulders and for (ii) dominant and non-dominant shoulders of the asymptomatic and symptomatic subjects, and, for the symptomatic and asymptomatic shoulders of the subjects with symptoms are presented in Table 2. The mean distances for the subjects without symptoms for these variables ranged from 5.9 to 6.3 cm and for the subjects with symptoms from 6.0 to 6.5 cm.

**Table 2:** Pectoralis minor length test distance

	**Mean (cm)**	**SD**	**Minimum (cm)**	**Maximum (cm)**
	
**Asymptomatic subjects**				
Mean of 3 measurements (left)	5.9	1.4	3.5	8.9
Mean of 3 measurements (right)	6.0	1.4	3.0	9.5
Mean of 3 measurements (dominant)	6.3	1.4	3.5	9.5
Mean of 3 measurements (non – dominant)	5.9	1.3	3.0	8.9
				
Mean of first measurement (left)	5.9	1.4	3.5	8.9
Mean of first measurement (right)	6.0	1.4	3.0	9.5
Mean of first measurement (dominant)	6.0	1.5	3.5	9.5
Mean of first measurement (non – dominant)	5.9	1.3	3.0	8.9
				
**Symptomatic subjects**				
Mean of 3 measurements (left)	6.2	1.5	2.9	10.1
Mean of 3 measurements (right)	6.4	1.4	2.8	9.8
Mean of 3 measurements (dominant)	6.3	1.4	2.8	9.8
Mean of 3 measurements (non – dominant)	6.0	1.4	2.9	10.0
Mean of 3 measurements (asymptomatic)	6.4	1.5	2.8	10.1
Mean of 3 measurements (symptomatic)	6.1	1.3	2.9	10.1
				
Mean of first measurement (left)	6.2	1.4	3.0	10.0
Mean of first measurement (right)	6.4	1.3	3.0	9.8
Mean of first measurement (dominant)	6.5	1.4	2.8	10.0
Mean of first measurement (non – dominant)	6.1	1.3	3.0	10.0
Mean of first measurement (asymptomatic)	6.4	1.5	2.8	10.0
Mean of first measurement (symptomatic)	6.2	1.3	3.0	9.8

Abbreviations: SD (standard deviation)

The ICC (2,1) and ICC (2,3) results together with the 95% CI and SEM results for the left and right shoulders of the subjects without symptoms are presented in Table 3 and in Table 4 for the subjects with symptoms. For the subjects without symptoms the ICC (2,1) results ranged from .92 to .93 and the ICC (2,3) results from .92 to .96. For the subjects with symptoms the ICC (2,1) results ranged from .90 to .93 and the ICC (2,3) results from .95 to .97.

**Table 3:** Asymptomatic subjects: ICC, 95% CI and SEM results

Measurement	Single measure ICC (2,1)	95% CI	SEM cm (mm)
Right shoulder (1^st ^measurement)	.92	.86 to .96	0.40 (4 mm)
Left shoulder (1^st ^measurement)	.93	.88 to .96	0.37 (4 mm)
Dominant shoulder (1^st ^measurement)	.93	.86 to .96	0.38 (4 mm)
Non dominant shoulder (1^st ^measurement)	.92	.87 to .96	0.39 (4 mm)

	Average measure ICC (2,3)	95% CI	SEM cm (mm)

Right shoulder (mean of 3)	.96	.93 to .98	0.29 (3 mm)
Left shoulder (mean of 3)	.92	.94 to .98	0.28 (3 mm)
Dominant shoulder (mean of 3)	.96	.94 to .98	0.29 (3 mm)
Non dominant shoulder (mean of 3)	.96	.93 to .98	0.28 (3 mm)

Abbreviations:

ICC (intraclass correlation coefficient), CI (confidence interval), SEM (standard error of measurement)

**Table 4:** Symptomatic subjects: ICC, 95% CI and SEM results

Measurement	Single measure ICC (2,1)	95% CI	SEM cm (mm)
Right shoulder (1^st ^measurement)	.90	.81 to .94	0.42 (5 mm)
Left shoulder (1^st ^measurement)	.92	.86 to .96	0.40 (4 mm)
Dominant shoulder (1^st ^measurement)	.92	.86 to .95	0.41 (5 mm)
Non dominant shoulder (1^st ^measurement)	.91	.84 to .95	0.40 (4 mm)
Painful side (1^st ^measurement)	.91	.84 to .95	0.45 (5 mm)
Painfree side (1^st ^measurement)	.93	.87 to .96	0.34 (4 mm)

	Average measure ICC (2,3)	95% CI	SEM cm (mm)

Right shoulder (mean of 3)	.95	.91 to .97	0.31 (4 mm)
Left shoulder (mean of 3)	.97	.94 to .98	0.26 (3 mm)
Dominant shoulder (mean of 3)	.95	.92 to .98	0.31 (4 mm)
Non dominant shoulder (mean of 3)	.96	.92 to .98	0.28 (3 mm)
Painful side (1^st ^measurement)	.95	.91 to .97	0.34 (4 mm)
Painfree side (1^st ^measurement)	.97	.94 to .98	0.23 (3 mm)

Abbreviations:

ICC (intraclass correlation coefficient), CI (confidence interval), SEM (standard error of measurement)

To determine the relationship between the pectoralis minor length test and symptoms a 2 × 2 table was constructed [33]. The results are reproduced in Table 5 which details how the variables were calculated.

**Table 5:** Sensitivity and specificity of the pectoralis minor length test

		**SUBJECTS**		
		
		***Subjects with symptoms of non-traumatic origin***	***Subjects/shoulders without symptoms***	**Totals**
**Diagnostic test result for the pectoralis minor length test (cm)**	**≥ 2.6**	***a* 45 **	***b* 90 **	*a+b ***135**
	**< 2.6**	***c *0**	***d *0**	*c+d ***0**
	**Totals**	*a+c ***45**	*b+d ***90**	*a+b+c+d ***135**

Abbreviations: ICC (intraclass correlation coefficient), CI (confidence interval), SEM (standard error of measurement)

The sensitivity, specificity and positive and negative likelihood ratios for the pectoralis minor length test are detailed in Table 6. The results suggest that the sensitivity of the test was 100%, the specificity was 0% the positive likelihood ratio was 1 and the negative likelihood ratio was indeterminable.

**Table 6:** Values for the diagnostic accuracy of the pectoralis minor length test.

**Sensitivity**	True positive rate/True positive rate and false negative rate	Cell A/Cell A and Cell C	**100%**
**Specificity**	True negative rate/True negative rate and false positive rate	Cell D/Cell D and Cell B	**0%**
**Positive Likelihood Ratio (LR+)**	Sensitivity/1-Specificity	100/100-0	**1**
**Negative Likelihood Ratio (LR-)**	1- Sensitivity/Specificity	100-100/0	**Indeterminable**

## Discussion

The findings of this investigation suggest that using the method employed in this study the pectoralis length test measurement demonstrated acceptable clinical intra-rater reliability. The single measure ICC (2,1) results ranged from .92 to .93 for the subjects without symptoms, and the comparable results for the subjects with symptoms ranged from .90 to .93. Portney and Watkins [35] has suggested that ICC values above .75 are indicative of good reliability and those below .75 should be considered as poor to moderate. Portney and Watkins [35] (page 565) state; "For many clinical measurements reliability should exceed .90 to ensure reasonable validity". All the ICC measurements in this investigation exceeded .90 which suggests they have exceeded the threshold for both good reliability and reasonable validity. The standard error of measurement (SEM) provides the clinician with an estimation of the error associated with a measurement in the units used to make that measurement. The SEM results for both the subjects with and without symptoms ranged from 3 mm to 5 mm. This finding suggests that there is approximately 0.5 cm error associated with the method used in this investigation to measure the pectoralis minor length test. From a clinical perspective 1 SEM indicates that the clinician may be 68% certain that the true measurement value lies between +/- 0.5 cm from the measured value. Two SEM provides the clinician with 95% confidence of the true pectoralis minor length test measurement value. The results of the current investigation suggest this value would lie within +/- 1.0 cm from the measured value.

The diagnostic value of this test was investigated by calculating the sensitivity and specificity of this test using the recommended 2.6 cm distance as the 'gold standard' reference measurement. Postural and muscle imbalance theory suggests that a short pectoralis minor is associated with a number of syndromes effecting the shoulder and upper quadrant. The pectoralis minor length test is one method that has been recommended to determine if this muscle is of normal length or is short and the 2.6 cm distance has been proposed as the length that separates a muscle of normal length to one that is short and may be associated with symptoms. The mean measurements ranged from 5.9 cm to 6.3 cm in the asymptomatic group and from 6.0 cm to 6.5 cm in the symptomatic group. These results compare closely to those reported by Borstad [36] who recorded mean pectoralis length test distance ranges from 5.96 cm to 6.57 cm in 50 asymptomatic subjects that had been subdivide into two separate groups. In the current investigation the pectoralis length test distance was measured in 90 subjects (or 180 shoulders) and the minimum distance recorded was 2.8 cm in the symptomatic group and 3 cm in the asymptomtic group. Both the mean and minimum measurements are greater than the stated 2.6 cm measurement that separates a muscle of normal length to one that is short. This finding, in this group of subjects, questions the basis of the 2.6 cm measurement as an appropriate clinical guideline and if used as recommended, clinicians will potentially implicate a pectoralis minor muscle imbalance in every patient assessed. This concern is reflected in the calculation of the sensitivity, specificity and likelihood ratios. Although the sensitivity for this test was found to be 100% which is considered ideal, the specificity was found to be 0%. The clinical implication of this is that every patient will be 'ruled in' as having a shortened pectoralis minor and no patient (even those without shoulder pathology) will be 'ruled out'.

Sahrmann [16] has recommended that in order to further test the length of pectoralis minor a stretch applied in a superior lateral direction should place the posterior border of the acromion against the table. However the assumption is that a linear distance greater than 2.6 cm should alert the clinician to the possibility of a tight pectoralis minor, and the findings of this study suggest that this assumption may not be relevant in the clinical reasoning process. It is possible that the stretch recommended by Sahrmann [16] may be of more value to the clinician than the measurement of the table to posterior acromion measurement. However before this is test is embraced clinically, the reliability, validity, diagnostic accuracy and clinical utility needs to be confirmed.

Although pectoralis minor shortening is central to the theory that is used to underpin the muscle imbalance approach in relation to shoulder and upper quadrant pathology the findings of this investigation suggest that although the test is reliable it lacks the diagnostic accuracy to identify subjects that have a shortening of this muscle as a cause or contribution to their symptoms. As such, at present, the findings of this investigation suggest this measurement should not be used as part of the clinical evaluation of patients with shoulder conditions. This finding supports the conclusions of Borstad [36] who reported that following a series of postural measurements in a group of 50 subjects without symptoms that the supine method of measuring pectoralis minor length appeared to lack validity. The current investigation suggests that this is also the case in subjects with shoulder symptoms. Borstad and Ludewig [37] proposed an alternative method for assessing the pectoralis minor length by measuring the distance from the caudal edge of the fourth rib at the sternum and the inferomedial aspect of the coracoid process. In a cadaver study (n = 11) Borstad and Ludewig [37] reported that the external measurement correlated with the length of pectoralis minor length following dissection. However, confounding variables such as postural sway and respiration cannot be controlled for in cadaveric investigations [38]. To normalize the measurement the pectoralis minor length Borstand and Ludewig [37] recommended dividing the measurement by the subject's height and multiplying by 100. Of relevance, Borstad and Ludewig [37] reported that asymptomatic subjects with a relatively shorter normalized pectoralis minor length demonstrated significantly less scapular posterior tilt at 90° and 120° sagittal plane, scapular plane and coronal plane humeral elevation. At present there is no evidence to suggest that this will lead to the development of shoulder pathology and a longitudinal study of symptom development in a sufficiently large group of asymptomatic subjects of varying normalized pectoralis minor lengths would contribute to the body of knowledge required to better understand the relationship between posture and symptoms.

The review of the literature did not identify any investigation that has demonstrated the validity of the supine measurement in subjects with and without symptoms. Although it is possible to visualise how a shortened pectoralis minor may decrease the pectoralis minor length test distance there are other possible reasons for this distance to vary, such as variations in the normal bony anatomy of the scapula or the rib cage. Although the clinical scenarios that have been proposed to implicate pectoralis minor shortening specifically and muscle imbalance generally are appealing and provide a framework for a clinician to guide the clinical evaluation and basis for treatment in patients with symptoms the available literature at best is supported by equivocal evidence.

A FHP is identified when the head sits forward of a plumb line that normally joins the tragus of the ear to the lateral malleolus of the ankle [13,28]. The plumb-line method for assessing posture is used to identify deviations from an ideal which are argued to be associated with muscle imbalance and increased joint stress and pathology. A FHP is identified when the tragus of the ear lays anterioly to the plumb line [13,28]. The basis for the existence of ideal posture has been challenged. Grimmer [29] examined FHP in 427 randomly selected subjects without symptoms. Subjects were examined in unconstrained sitting using a custom built Linear Excursion Measuring Device. The plumb-line measurement described by Kendall et al [13,28] was defined as the baseline for ideal posture. No subject was found to have a resting FHP perfectly aligned with the ideal norm (vertical reference line).

Lewis et al [30] investigated the relationship between forward head posture, thoracic kyphosis, range of shoulder flexion and abduction range and, selected positions of the scapula in 60 subjects without symptoms and 60 subjects with a clinical diagnosis of subacromial impingement syndrome. The findings of their investigation suggested that upper body posture does not follow the set patterns of postural change and muscle imbalance described in the literature, and concluded that further research is required to determine if upper body, scapular posture, and muscle imbalance are involved in the pathogenesis of subacromial impingement syndrome. This finding supported those of Raine and Twomey [26] who reported that no relationship existed between forward head posture, forward shoulder posture and thoracic kyphosis.

The posture of 30 subjects with shoulder overuse injuries was compared with an age and gender matched group of 30 subjects without symptoms [23]. No difference in posture was reported between the groups for the thoracic kyphosis angle as well as scapular protraction and rotation. Although this study did not find support for the contention that a relationship between posture and pathology exists, a number of confounding factors including the method used for measuring scapular rotation may have influenced the findings.

Other studies that have reported equivocal findings relating to posture, scapular position and muscle function include investigations of subjects without symptoms [12,22] as well as with and without subacromial impingement syndrome [25,39]. These studies have reported findings that have both supported and diverged from theories associated with posture and muscle imbalance.

The conclusion of all these studies is that further research is required to more fully understand the relationship between posture and function around the shoulder and upper quadrant aiming to determine if a relationship between posture and muscle imbalance exists and if it does are the changes that are identified the cause or the result of pathology and pain. In addition to this, tests that measure these changes in posture and muscle imbalance that are both reliable and have diagnostic accuracy need to be identified.

Another syndrome associated with a tight pectoralis minor is the scapular downward rotation syndrome [16]. The assumption is that increased scapular downward rotation is a posturally abnormal position and considered to be a cause or mechanism for the perpetuation of shoulder symptoms. In support of this, Basmajian and Bazant [40] have described that when correctly orientated the glenoid fossa of the scapula should face superiorly. Although no explanation was provided how the correct orientation was determined, Basmajian and Bazant [40] argued that a superior inclination was important to provide a bony support for the humeral head. However, and in contrast to this the findings of two radiological studies [41,42] have described that normally the glenoid fossa displays a downward inclination. In addition to this, the position of the scapula was measured in 20 asymptomatic subjects before and after a 6 week muscle strengthening and stretching program [43]. Following the program the scapula was reported to be in greater downward rotation at different levels of shoulder elevation and this was associated with an overall greater range of shoulder abduction following the exercise program. To some extent this finding challenges the belief that increasing the upward rotation of the scapula is essential to restore normal function and again suggests considerably more research is required on the importance of scapular position and its potential relationship between function and pathology.

It is acknowledged that in the current study diagnostic categories of shoulder pathology were mixed. However, postural theory involving the identification of a muscle imbalance, such as a short pectoralis minor does not restrict itself to only one type of pathology, and the assumption is that if an imbalance is identified it should be corrected as it has been postulated that the imbalance may cause or contribute to the presenting symptoms. Identification is only possible when the test proposed to identify the postural anomaly is reliable. However, any clinical test of posture and muscle imbalance must also be able to differentiate patients whose symptoms relate to that imbalance and postural variation to those whose symptoms do not relate to their posture. In the context of the pectoralis minor length test, as it is currently described in the literature, the findings of the present study suggest that a clinician cannot confidently use the information derived from the test to determine the involvement of the muscle with the presenting symptoms.

## Conclusion

The pectoralis minor length test has been advocated to clinically identify a shortening of this muscle that may be associated with clinical syndromes, loss of function and pain in the shoulder and upper quadrant. The findings of this investigation suggest that the pectoralis minor length test is a reliable method to measure the distance from the treatment table to the posterior aspect of the acromion. However the recommended 'gold standard' reference of a normal distance of 2.6 cm is not supported in this investigation. Subjects without symptoms and subjects with shoulder symptoms were found to have a mean distance from the table of approximately 6 cm, over twice the recommended distance. This finding and the analysis of the diagnostic utility of this test (sensitivity, specificity, positive and negative likelihood ratios) suggest that the evaluation procedure as currently advocated lacks diagnostic value and clinicians using this test to inform the clinical reasoning process in terms of delineating the pathogenesis and aetiology of the symptoms, help in treatment planning and explanations given to patients must do so with caution.

Although the theories used to underpin the muscle imbalance and postural model are appealing this investigation has highlighted that further research is required to determine the validity of this model together with the reliability of the clinical evaluation procedures and the diagnostic accuracy of the tests. It is acknowledged that the 100% of the asymptomtic subjects in this investigation that were found to have a pectoralis minor length test distance of more than 2.6 cm may develop shoulder and upper quadrant symptoms at some stage in the future. However, the possibility of this would need to be investigated in a longitudinal study on a group of asymptomic subjects. As such clinicians need to carefully consider how to interpret the findings of clinical examinations of posture and muscle imbalance when clinically reasoning the cause of pathology and how best to manage the individual patients condition.

## Competing interests

The author(s) declare that they have no competing interests.

## Authors' contributions

Both authors contributed to the design, data collection and analysis of this investigation. Both authors have read and approved the final manuscript.

## Pre-publication history

The pre-publication history for this paper can be accessed here:


